# De Novo Generation of Singlet Oxygen and Ammine Ligands by Photoactivation of a Platinum Anticancer Complex**

**DOI:** 10.1002/anie.201307505

**Published:** 2013-10-25

**Authors:** Yao Zhao, Nicola J Farrer, Huilin Li, Jennifer S Butler, Ruth J McQuitty, Abraha Habtemariam, Fuyi Wang, Peter J Sadler

**Affiliations:** Department of Chemistry, University of Warwick, CV4 7AL (United Kingdom); Beijing National Laboratory for Molecular Sciences, CAS Key Laboratory of Analytical Chemistry for Living Biosystems, Institute of Chemistry, Chinese Academy of Sciences, Beijing 100190 (P.R. China)

**Keywords:** antitumor agents, bioinorganic chemistry, guanine oxidation, photoactivation, singlet oxygen

The potential for spacial selectivity, as offered by photoactivation, together with novel excited-state chemistry and accompanying mechanisms of action make exploration of photoactivated metal chemotherapeutic complexes attractive for cancer therapy.^1^We have been studying Pt^IV^-diazidodihydroxido anticancer complexes, [Pt(N_3_)_2_(OH)_2_(Am_1_)(Am_2_)] (Am_1_/Am_2_=am(m)ines),^2^ which, in the absence of light, exhibit minimal cytotoxicity towards cancer cells and do not react with glutathione (GSH), 5′-guanosine monophosphate (5′-GMP), or DNA in either cell-free media or aqueous solutions. By contrast, upon irradiation with UVA or visible (blue/green) light, these complexes display potent cytotoxicity towards a range of cancer cell lines. The reported photodecomposition products include azide anions (N_3_^−^), azidyl radicals (N_3_^.^), nitrogen gas (N_2_), and oxygen gas (O_2_).^3^ A particularly potent photocytotoxic anticancer complex is *trans*,*trans*,*trans*-[Pt(N_3_)_2_(OH)_2_(MA)(Py)] (**1**, MA=methylamine, Py=pyridine).^4^ Herein we report the unprecedented oxidation of 5′-GMP by **1** upon irradiation with UVA and the identification of some unexpected reaction pathways involving singlet oxygen (^1^O_2_) and nitrene (Pt-N) intermediates. The source of the ^1^O_2_ was also investigated.

The photoreaction of **1** with 5′-GMP upon irradiation with blue light with a wavelength of 450 nm for 1 h gave (*SP*-4-2)-[Pt(N_3_)(MA)(Py)(5′-GMP)]^+^ (**1 a**^+^) as the major product and *trans*-[Pt(MA)(Py)(5′-GMP)_2_]^2+^ (**1 b**^2+^) as the minor product (Figure 1 A). A similar result has been reported for **1** under slightly different conditions, and for related compounds.2a, 4, 4 However, when the reaction mixture was irradiated at 420 nm for 30 min, two new photoproducts, **1 c** and **1 e**, were observed by HPLC (Figure 1 B). When the sample was irradiated with UVA (365 nm) for 15 min, one more species, **1 d**, was found (Figure 1 C). The isotopic distributions observed in the ESI-MS analysis revealed that the singly charged cations of **1 c** (*m*/*z*=718.1), **1 d** (*m*/*z*=684.1), and **1 e** (*m*/*z*=700.1) all contain a Pt atom. A control experiment with 5′-GMP in the absence of **1** and UVA irradiation for 15 min showed no reaction (Figure 1 D), thus suggesting that 5′-GMP is stable under these conditions. The photoreaction of **1** and 5′-GMP under an atmosphere of argon gave similar results, thereby excluding the possibility that dissolved oxygen is the oxidant. Hence, **1 c**, **1 d**, and **1 e** are all produced by the photoreaction of **1**.

**Figure 1:**
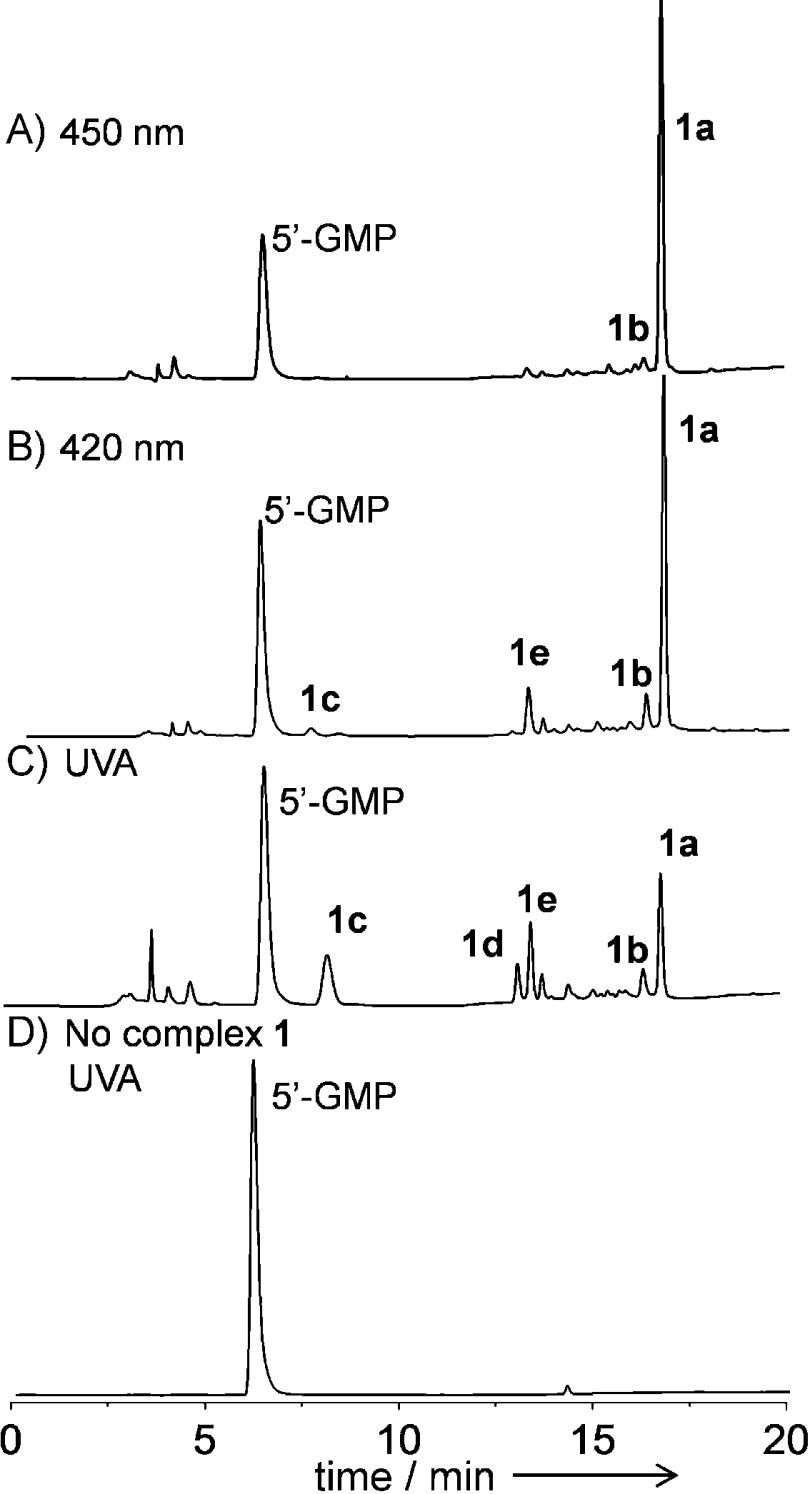
Chromatograms for the photoreactions of 1 (0.67 mm) with 5′-GMP (1.0 mm) in aqueous solution upon irradiation with A) 450 nm light, 50 mW cm^−2^, 60 min; B) 420 nm, 4.3 mW cm^−2^, 30 min; C) UVA (365 nm), 3.5 mW cm^−2^, 15 min; D) 5′-GMP (1.0 mm) only, UVA, 15 min.

High-resolution MS of **1 a**, **1 c**, **1 d**, and **1 e**, as well as their tandem MS (CID), were performed to examine further the structure of each species (see Figures S1 and S2 in the Supporting Information). They are all assigned as Pt^II^ complexes, and their structures are listed in Table 1, while fragment ions are listed in Tables S1–S4 in the Supporting Information, together with their assignments.

**Table 1:** Positive ions for complexes 1 a, 1 c, 1 d, and 1 e observed by HRMS and the corresponding assignments.

Found *m*/*z*	710.1205	718.1337	684.1228	700.1235
Proposed chemical structure	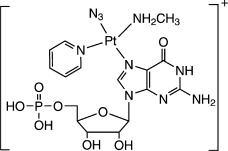	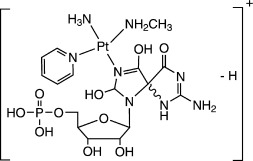	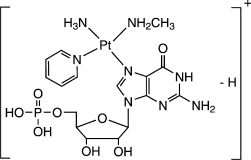	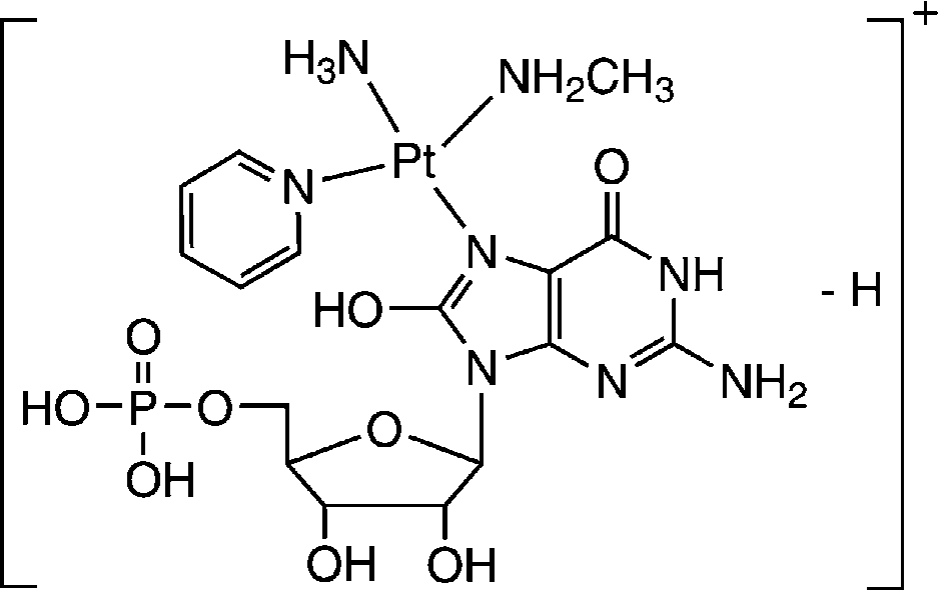
	([**1 a**]^+^)	([**1 c**−H]^+^)	([**1 d**−H]^+^)	([**1 e**−H]^+^)
Theoretical *m*/*z*	710.1164	718.1314	684.1260	700.1209
Error [ppm]	5.8	3.2	4.7	3.7

Curiously, species **1 c**, **1 d**, and **1 e** all have an NH_3_ ligand, which was not present in the reactants. A reasonable source of the NH_3_ is the {Pt-N_3_} fragment, which can lose N_2_ upon irradiation with light to form a {Pt-N} nitrene intermediate.^6^ This postulation was verified by using 1*, azide *trans*,*trans*,*trans*-[Pt(N_3_*)_2_(OH)_2_(MA)(Py)], where N_3_*=[^15^N=^14^N=^14^N], and hence each bound N atom from the azide is now 50 % ^15^N. The photoreaction of 1* with 5′-GMP, carried out under identical conditions, gave an identical chromatogram as that shown in Figure 1 C. The ESI-MS spectra for 1 a*, 1 c*, 1 d*, and 1 e*, which have the same retention times as **1 a**, **1 c**, **1 d**, and **1 e**, are shown in Figure S5 in the Supporting Information. The molecular weight of compound 1 a* was 1 Da larger than **1 a**, thus suggesting that it has an intact N_3_*^−^ ligand. The isotope distributions of 1 c*, 1 d*, and 1 e* indicate that they are all 50 %/50 % mixtures of [*M*] and [*M*+1], so they are all considered as {Pt-NH_3_*} fragments derived from {Pt-N_3_*} (NH_3_*=50 % ^15^NH_3_/50 % ^14^NH_3_). This result suggests that N_2_ gas is released directly from {Pt-N_3_}, thereby generating a {Pt-N} intermediate.

The release of N_2_ on photolysis of **1** was verified by ^14^N NMR spectroscopy (see Figure S6 in the Supporting Information); signals for free azide (N_3_^−^) were also detected (see the Supporting Information for details). N_2_ may be released directly from {Pt-N_3_} and also may be formed from the recombination of the azidyl radicals (N_3_^.^) generated in this photoreaction.^7^ The release of N_3_^.^ was confirmed by EPR spectroscopy by using 5,5-dimethylpyrroline-*N*-oxide (DMPO) as the spin trap. Signals for the DMPO-^14^N_3_ spin adduct were detected (see Figure S7 in the Supporting Information), but hydroxyl radicals OH^.^ were not trapped. It is evident that 5′-GMP is not oxidized by N_3_^.^, as its presence did not affect the trapping of N_3_^.^ radicals (see the Supporting Information for details).

Species **1 e** contains an 8-hydroxyguanine (8-OH-G) fragment, equivalent to 8-oxoguanine (8-oxo-G), which is one of the most common products of DNA oxidation.^8^ The possibility that the oxidation of guanine by photoactivated **1** involved singlet oxygen or nitrene intermediates was investigated. Product **1 c** has a similar structure as **1 e**, but the 8-OH-G is replaced by RedSp (*N*-formylamidoiminohydantoin),8a hydrolyzed 8-OH-G (shown in Scheme S1 in the Supporting Information).

During the photoreaction of **1** and 5′-GMP with irradiation at 450 nm the yellow color of the solution became darker, and gas bubbles formed (see Figure S8 in the Supporting Information). Similar results were obtained when the reaction was triggered with UVA. The evolution of the gases O_2_ and N_2_ was verified by GC-MS performed in ^18^O-labeled water under an argon atmosphere, with the aim also of examining the source of the O atoms in the generated O_2_. After the photolysis of **1**, the gas phase was analyzed by GC-MS (see Figure S9 and the Supporting Information for details), and ^16^O_2_ (*m*/*z*=32) and N_2_ (*m*/*z*=28) were both found. No ^18^O-substituted O_2_ was detected. This result verified the release of N_2_ and O_2_ and, moreover, provided evidence that the two oxygen atoms in the generated O_2_ are both from **1** rather than from the solvent.

The nature of the released oxygen was investigated using a fluorescence probe for singlet oxygen: SOSG. SOSG is a highly selective sensor for ^1^O_2_ without any appreciable response to hydroxyl radicals or superoxide.^9^ In the absence of ^1^O_2_, SOSG exhibits low fluorescence, but in the presence of ^1^O_2_, strong green fluorescence can be observed with *λ*_ex_=504 nm and *λ*_em_=525 nm. Solutions containing **1** and SOSG were stable in the dark or even upon irradiation at *λ*≥504 nm. However, when exposed to weak irradiation at 365 nm (21 μW cm^−2^), the intensity of the fluorescence at 525 nm increased rapidly, thus indicating that ^1^O_2_ was generated (Figure 2). The dose-dependent efficiency of generating ^1^O_2_ upon irradiation was higher the shorter the wavelength (UVA>420 nm>450 nm, see Figure S10 in the Supporting Information). Control experiments carried out in the dark or in the absence of **1** showed no change in the fluorescence intensity (see Figure S10 in the Supporting Information). A sample saturated with argon was irradiated at 365 nm and gave stronger fluorescence (Figure 2). This result revealed that the ^1^O_2_ was not generated from the dissolved O_2_ through energy transfer from a photosensitizer. N_2_ had a similar effect as argon. Neither argon nor N_2_ itself could trigger the fluorescence of SOSG. The release of singlet oxygen from a Pt^IV^-diazidodihydroxido complex upon irradiation with light in the absence of any exogenous source of oxygen gas appears to be unprecedented.

**Figure 2:**
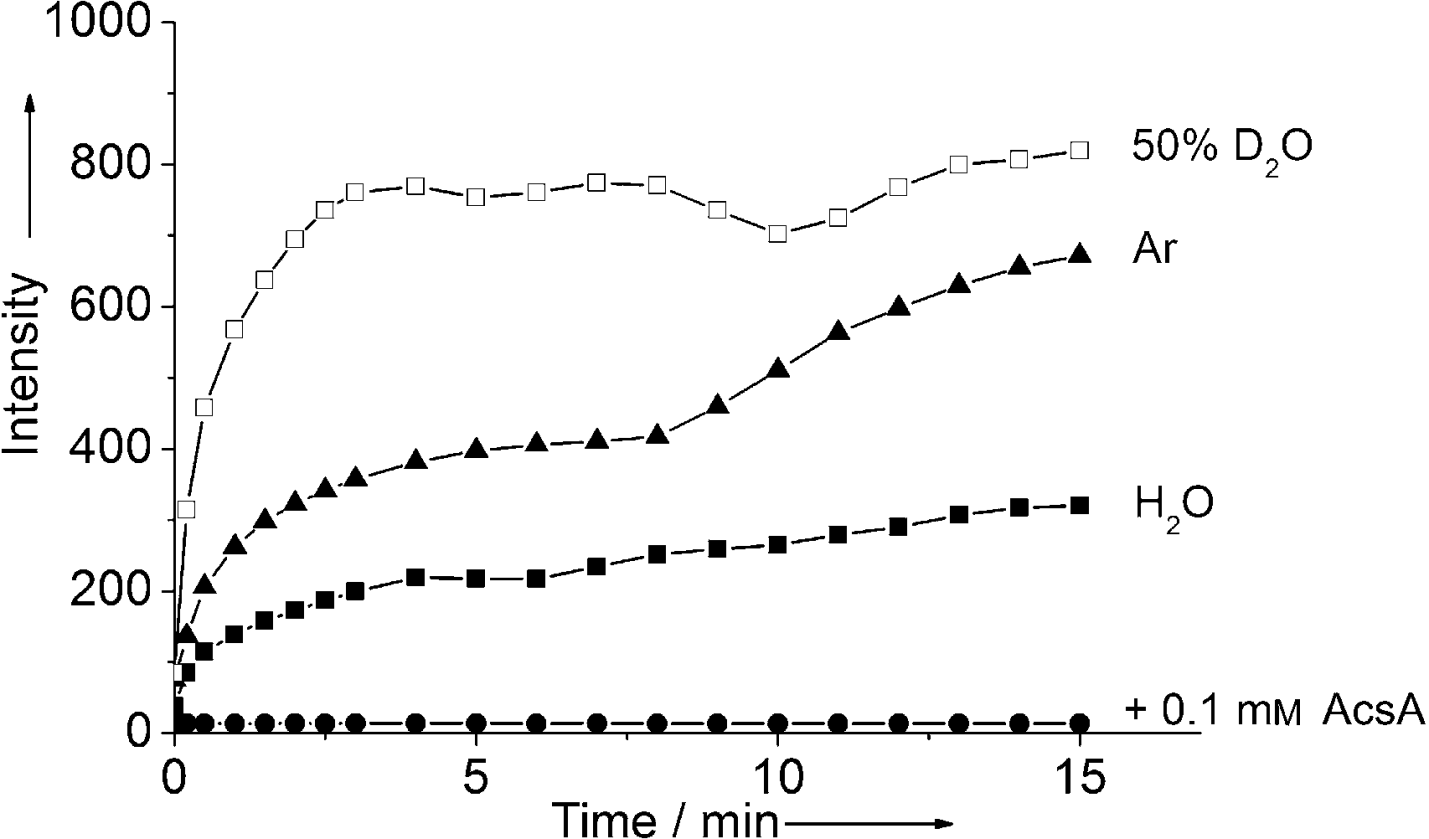
Time-dependent fluorescence (*λ*_ex_/*λ*_em_=504/525 nm) from 1 (50 μm) and SOSG (1 μm) in H_2_O (3 % MeOH) upon weak irradiation at 365 nm (21 μW cm^−2^) (293 K). ▪: no additive; □: 50 % D_2_O; ▴: saturated with argon; •: 0.1 mm l-ascorbic acid (AscA). All the data points were the average of 2–4 independent experiments.

The lifetime of ^1^O_2_ in D_2_O is known to be much longer than that in H_2_O.^10^ Indeed, the fluorescence intensity arising from the reaction of **1** with SOSG in 50 % D_2_O with *λ*_irr_=365 nm was three- to fourfold higher than that of the reaction carried out in H_2_O alone (Figure 2). The reaction was repeated in the presence of 100 μm l-ascorbic acid (AscA) as a ^1^O_2_ scavenger. Complex **1** does not react with AscA in the absence of light, even though AscA is a strong reductant. However, upon irradiation at 365 nm, the fluorescence was totally quenched (Figure 2). These results again confirmed the generation of ^1^O_2_.

Guanine and the other nucleobases may be oxidatively damaged by reactive oxygen species (ROS), radicals, and ionizing/UVA radiation.^11^ The oxidation of guanine usually leads to DNA damage so as to cause lethality, aging, and mutagenicity.^8^ Although it has been reported that several Pt^IV^-tetrachlorido complexes can directly oxidize guanine,^12^ we report here for the first time that the photodecomposition of a Pt^IV^-diazidodihydroxido complex can oxidize guanine. Complexes containing Pt^II^ and oxidized guanine as 8-OH-G and RedSp were detected (Table 1).

Two pathways can be proposed for the oxidation of guanine. The first involves a nitrene intermediate (Scheme 1, Mechanism 1). This can arise from loss of N_2_ from the {Pt-N_3_} fragment upon irradiation with UVA.^6^ Two electrons are transferred from guanine to the nitrene, and the guanine itself is oxidized to give 8-OH-G on addition of H_2_O. The nitrene is reduced and finally forms the ammine adduct {Pt-NH_3_}. Another possible oxidant is singlet oxygen. The ^1^O_2_ generated by photolysis of **1** can oxidize guanine to 8-OH-G by a direct [4+2] cycloaddition (Scheme 1, Mechanism 2).8a

**Scheme 1:**
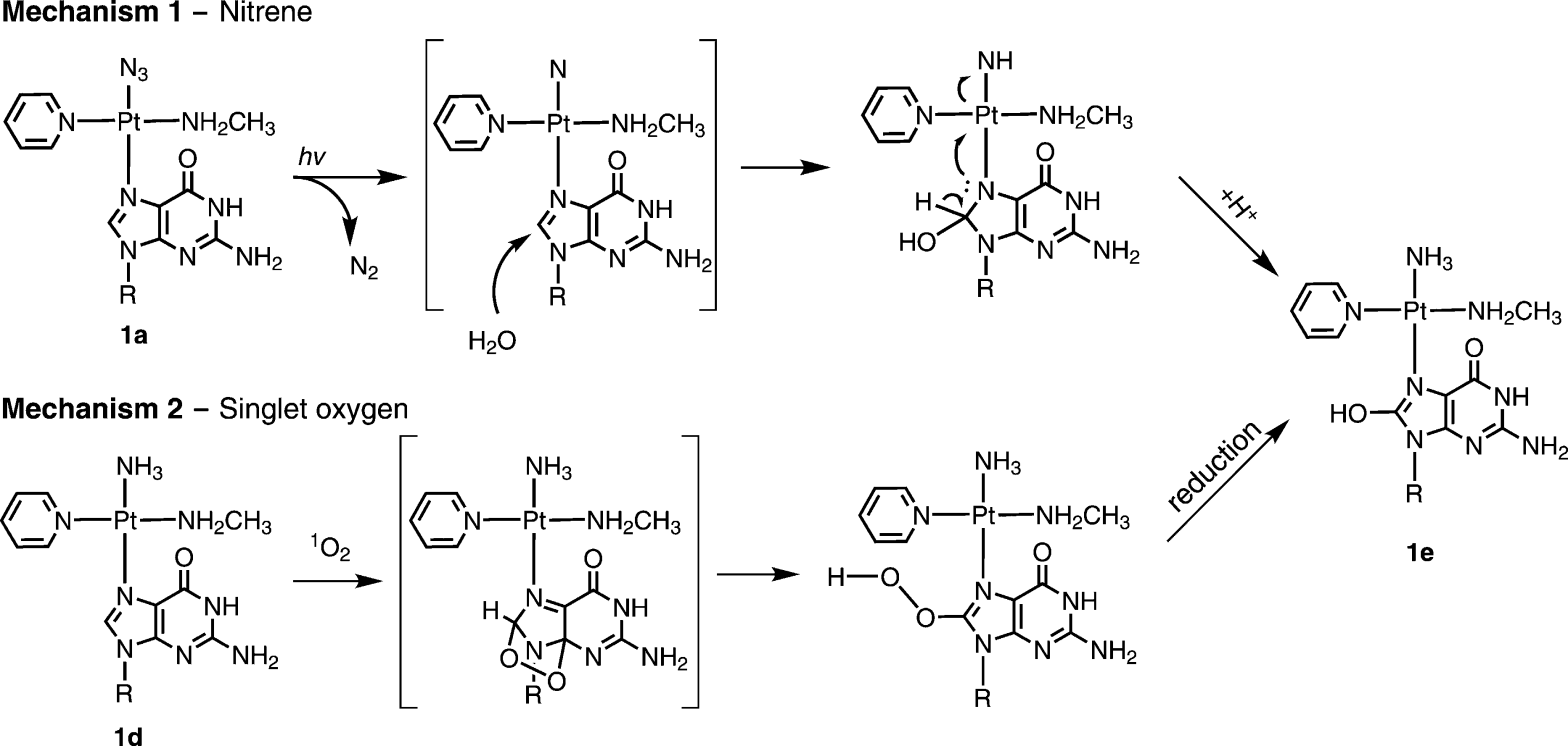
Two possible mechanisms for the oxidation of 5′-GMP. Charges are omitted for clarity.

Singlet oxygen (^1^O_2_) is a highly reactive and toxic species in biological systems. It is considered the principal antiproliferative species in photodynamic therapy (PDT), in which it reacts with many biomolecules, including nucleic acids, proteins, and lipids, thereby causing cancer cell death.^8^, ^13^ A common method to generate ^1^O_2_ is photosensitization, which produces ^1^O_2_ by energy transfer from a photoexcited sensitizer to ground-state triplet oxygen (^3^O_2_). This strategy is the basis of current PDT, but requires the presence of oxygen at the target site. However, tumor cells are often hypoxic. In contrast, the generation of ^1^O_2_ from *trans*,*trans*,*trans*-[Pt(N_3_)_2_(OH)_2_(MA)(Py)] upon irradiation with UVA/blue light does not require any exogenous source of oxygen gas (Figure 2). This feature may be beneficial for the potential clinical application of **1** and killing of hypoxic cancer cells. Moreover, the wavelength can be tuned to control its photocytotoxicity, as shorter wavelengths generate more ^1^O_2_.

Since neither of the oxygen atoms in the released ^1^O_2_ originate from water, the most plausible source is the OH groups of **1**. Furthermore, solvent substitution at Pt^IV^ is not likely to occur before its reduction to Pt^II^. A possible photolysis mechanism is given in Scheme 2. Upon irradiation with short-wavelength light, such as UVA, photodecomposition of **1** in the presence of 5′-GMP may occur through two pathways. In the first, two azidyl radicals (N_3_^.^) are lost and Pt^IV^ is reduced to Pt^II^. The OH groups are protonated and thus are substituted by 5′-GMP to give product **1 b**. The second pathway is more complicated, but perhaps more likely. The integration of the HPLC peaks in Figure 1 C suggested that about 92 % of the Pt species were produced through this pathway. Complex **1** loses one azide ligand (N_3_^−^) and the OH ligands donate one electron each to reduce the Pt^IV^ to Pt^II^; they then rapidly recombine to generate oxygen gas, as singlet oxygen, through H_2_O_2_ formation.^14^ Then the binding of 5′-GMP to Pt produces **1 a**, which can be further transformed to **1 e** by loss of N_2_ gas from the N_3_ ligand, as in Scheme 1. Alternately, if N_2_ is expelled from the N_3_ ligand before 5′-GMP binds, **1 d** is produced, which can also be transformed to **1 e** by reacting with ^1^O_2_ (Scheme 1).

**Scheme 2:**
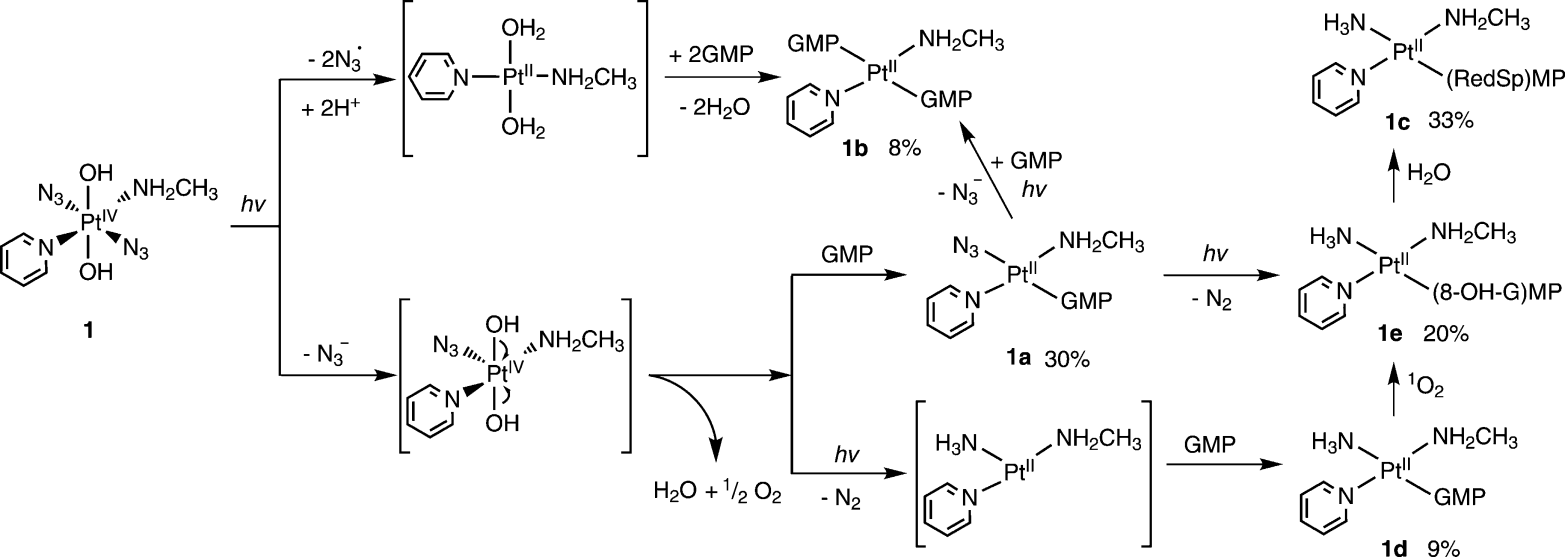
Possible mechanisms for the photoreaction of 1 with 5′-GMP upon irradiation with UVA. Species in square brackets are unstable intermediates. Charges are omitted for clarity. Species percentages are average HPLC integrations for four experiments with UVA irradiation (Figure 1 C).

There are a number of reports of the chemical reduction of Pt^IV^ to Pt^II^, and it is widely accepted that a concerted two-electron transfer from, for example, ascorbate, GSH, or guanine, to Pt^IV^ is involved.^12^, ^15^ However, the photoreduction of **1** may not follow the above pathway. Pt^IV^ is more likely to gain one electron from each of the two N_3_ or two OH ligands and give rise to N_3_^.^ or OH^.^ radicals, respectively. We were not able to trap OH^.^ radicals, perhaps because their lifetime is too short.

The in situ formation of an NH_3_ ligand can give rise to potential hydrogen-bonding interactions with DNA. DNA adducts of the type **1 d**, for example, may strongly inhibit RNA polymerase II and nucleotide excision repair.^16^ Nitrenes are highly reactive intermediates and are reported to be responsible for a wide range of DNA lesions.^17^ N_3_^.^ is a relatively mild and selective oxidant that can oxidize amino acids such as tryptophan.3b N_3_^−^ is a mitochondrial inhibitor, and a myeloperoxidase and catalase inhibitor. These species, together with ^1^O_2_, could all contribute to the potent photo-antiproliferative effect of **1** on cancer cells.

In summary, we observed the unexpected oxidation of guanine during the photoreaction of complex **1** (*trans*,*trans*,*trans*-[Pt(N_3_)_2_(OH)_2_(MA)(Py)]) with 5′-GMP. The photodecomposition of **1** involves Pt-nitrene intermediates and formation of singlet oxygen, free azide, azidyl radicals, and nitrogen gas. The oxidation of guanine is likely to arise from reactions of singlet oxygen and nitrene intermediates. The generation of singlet oxygen in the absence of oxygen gas and the oxidative damage to guanine may contribute to the potent photocytotoxic effects of this complex.

## Supporting Information

As a service to our authors and readers, this journal provides supporting information supplied by the authors. Such materials are peer reviewed and may be re-organized for online delivery, but are not copy-edited or typeset. Technical support issues arising from supporting information (other than missing files) should be addressed to the authors.

miscellaneous_information
