# Supplementary material for: De Novo Generation of Singlet Oxygen and Ammine Ligands by Photoactivation of a Platinum Anticancer Complex
Source: Angew Chem Int Ed Engl. 2013 Oct 25;52(51):13633–7. doi: 10.1002/anie.201307505 (PMC4230391; doi:10.1002/anie.201307505)
Supplement: Supplementary file 1 — miscellaneous_information [file anie0052-13633-sd1.pdf]

Supporting Information

© Wiley-VCH 2013

69451 Weinheim, Germany

De Novo Generation of Singlet Oxygen and Ammine Ligands by Photoactivation of a Platinum Anticancer Complex**

Yao Zhao, Nicola J. Farrer, Huilin Li, Jennifer S. Butler, Ruth J. McQuitty, Abraha Habtemariam, Fuyi Wang, and Peter J. Sadler**

anie_201307505_sm_miscellaneous_information.pdf

Supporting Information

Experimental details

Tables S1 – S4

Scheme S1

Figures S1 – S10

Experimental details

All chemicals used were obtained from Sigma-Aldrich or Fisher Scientific and used as received without any further purification, if unspecified. The water used in the experiments was deionised water from a Millipore water purification system. ^{15}N-labelled NaN_3 (1-^{15}N, 99%) was obtained from Cambridge Isotopes Lab, ^{18}O-water (97 atom %) from Aldrich, 5, 5-dimethyl-pyrroline-N-oxide (DMPO) (≥ 98%) from Enzo Life Sciences. Singlet Oxygen Sensor Green (SOSG) was obtained from Molecular Probes®, Inc. Photochemical reactions of Pt complexes with or without 5'-GMP were carried out at 298 K by using a LZC-ICH2 photoreactor (Luzchem Research Inc.) equipped with a temperature controller and 8 UVA lamps (Hitachi, λ_{max} = 365 nm, typically 3.5 mW/cm^2) with no other sources of light filtration. ACULED® VHL™ LEDs were also used (λ_{max} = 450 nm, typically 50 mW/cm^2) for irradiation of the samples.

NMR spectra were recorded on either a Bruker AV-600 (^{1}H, 600.13 MHz; ^{13}C, 150.9MHz; ^{195}Pt, 129.4 MHz; ^{15}N, 60.8 MHz; ^{14}N, 43.4MHz). Experimental parameters for ^{14}N NMR spectra (43.36 MHz): pulse sequences zgpg30 (proton broadband decoupled) was used and the digitization mode was in "baseopt" for improvement of baselines. The spectra were acquired with a relaxation delay (D1) of 0.25 s. Typical parameters were 32k data points and 64k scans. Data were processed using a line broadening of 20 Hz for EM window function unless otherwise stated. NH_4Cl (1.5 M) in 1 M HCl was used as an external ^{14}N reference (δ = 0); the chemical shift range between −100 and 500 ppm was scanned. The chemical shift observed in this way can be converted to CH_3NO_2 as reference, which is recommended by IUPAC,[1] by subtracting 380.5 ppm.

ESI-MS was performed on a Bruker Esquire 2000 mass spectrometer, HPLC-ESI-MS on a Bruker HCT-Ultra mass spectrometer coupled with an Agilent 1200 HPLC system with an Agilent ZORBAX Eclipse Plus C18, 5 μm, 4.6 × 250 mm column. Flow rate, 1.0 ml/min; detecting wavelength, 254 nm; mobile phase A, H_2O with 0.1% formic acid (FA); mobile phase B, MeOH with 0.1% FA. A linear gradient from 5% to 55% B over 15 min was applied to separate the photoreaction mixtures of Pt complexes with GMP. ESI-HR-MS and CID (collision-induced dissociation) tandem MS was performed on a Bruker MaXis UHR-Qq-TOF high resolution ESI-MS

spectrometer in positive-ion mode. A sample of formic acid was used as external calibration with linear mode. Samples containing 50.0 μM Pt were prepared in 20% H_2O/80% methanol.

The intensity of fluorescence was measured on a Jasco FP-6500 fluorimeter. The output light was used for both excitation and irradiation by adjusting to the wavelength required. The excitation and the emission slit widths were set to 3 nm. Filters were used to eliminate second order diffraction of shorter wavelengths from the specified longer wavelength monochromatic light. Power of individual irradiation wavelengths: 365 nm, 21 μW/cm^2; 420 nm, 0.85 mW/cm^2; 450 nm, 0.80 mW/cm^2. Fluorescence response time was 0.1 second and sensitivity was medium. Power levels were measured with an International Light Technologies Powermeter (ILT1400-A) equipped with a SEL033 detector and either a UVA/TD filter (315-390 nm) for UVA or a flat response visible filter F/W (400-1064 nm) for visible light. The sample of complex **1** (50 μM) and SOSG (1 μM) was prepared in deionized water with 3% methanol. MeOH was added to maintain the solubility of SOSG. The intensity of fluorescence was measured as soon as the irradiation stopped, where λ_{ex} = 504 nm and λ_{em} = 525 nm.

GC-MS was performed on a Shimadzu GCMS-QP2010 spectrometer. Complex **1** was dissolved in ^{18}O-water (10 mM) in a 2 mL sealable HPLC vial with Agilent screw cap and saturated with argon. The vial was sealed immediately and GC-MS was performed by injecting the gas in the vial, where only trace O_2 (O_2^+ at *m/z* 32) and N_2 (N_2^+ at *m/z* 28) were found. Then the sample was irradiated with UVA (λ_{max} = 365 nm, 4 mW/cm^2) for 0.5 h, and the gas in the vial was injected for GC-MS analysis again. The content of oxygen and nitrogen was substantially increased as indicated by their integrations relative to argon (Ar^+ at *m/z* 40).

The EPR spectra were recorded on a Bruker EMX (X-band) spectrometer. The ambient temperature experiments (*ca*. 293 K) were carried out using quartz tubes (1.0 mm ID × 1.2 mm O.D) obtained from Wilmad Labglass. A 50 μL Hamilton HPLC syringe needle was used to fill the quartz tubes, sealed with T-Blu Tac®. Typical instrument settings were: modulation amplitude 2.0 G and microwave power 0.63 mW. An aqueous solution of sample (5 mM) was added to an excess of spin-trap, DMPO (5, 5-dimethyl-pyrroline-N-oxide, 10 mM). Known concentrations of the EPR

standard TEMPOL (4-hydroxy-2,2,6,6-tetramethylpiperidin-1-oxyl) were used to obtain a standard calibration curve. From this curve, the concentration of spin adduct was determined. A 450 nm LED was used as the source of irradiation at a distance of *ca*. 30 cm from the EPR cavity. Samples were irradiated for 2 h and signals were recorded every 5 min. Controls of both DMPO and sample, irradiated and non-irradiated, were run as reference spectra. EPR spectra were analysed using Bruker WINEPR software and simulations were run using the SIMFONIA.

Trans,trans,trans-[$Pt(N_3)_2(OH)_2(MA)(Py)$] (**1**) was synthesized according to the published method.[2]

Caution! While no problems were encountered during this work, heavy metal azides are known to be shock and light sensitive, therefore it is essential that any Pt azido compound is handled with care. All syntheses were carried out under controlled (dim) lighting conditions.

The ^{15}N-N$_3$ labelled *trans,trans,trans*-[$Pt(N_3^*)_2(OH)_2(MA)(Py)$] (**1***) was synthesized by the same method as for **1** except the use of ^{15}N-labelled NaN_3 (99% Na[^{15}N=^{14}N=^{14}N]). ^{1}H NMR (600 MHz, 90% H_2O/10% D_2O, pH~5): δ = 8.74 (dd, $\underline{H_{2,6}}$, ^3J (^{1}H, ^{1}H) = 7 Hz, ^3J (^{195}Pt, ^{1}H) = 23 Hz, 2H), 8.23 (t, ^3J (^{1}H, ^{1}H) = 8 Hz, $\underline{H_4}$, 1H), 7.77 (t, ^3J (^{1}H, ^{1}H) = 7 Hz, $\underline{H_{3,5}}$, 2H), 6.14 (br, N$\underline{H_2}$, 2H), 2.40 (td, ^3J (^{1}H, ^{1}H) = 6 Hz, ^3J(^{195}Pt, ^{1}H) = 29 Hz, C$\underline{H_3}$, 3H). ^{195}Pt NMR (D_2O, 129.4 MHz): δ = 898.8 ppm. ^{15}N NMR (D_2O, 60.8 MHz): δ ($\underline{N_\gamma}$) = 165.6 ppm ($^3J_{Pt\text{-}N}$ = 24 Hz); δ ($\underline{N_\alpha}$) = 51.4 ppm ($^1J_{Pt\text{-}N}$ = 222 Hz). ESI-MS: $[M+Na]^+$ (*m/z*) calc., 448.1; found, 448.0. The purity was determined by an HPLC analysis to > 97%.

Synthesis of *Trans*-[$Pt(MA)(Py)(H_2O)_2$](BF_4)$_2$ (**1g**). *Trans*-[$Pt(Cl)_2(MA)(Py)$] (0.85 mg) was suspended in 1 mL H_2O, and $AgBF_4$ (8.7 mg, 2.1 mol equiv) was added and stirred at 333 K for 24 h. AgCl precipitate was filtered off using an inorganic membrane filter (Sartorius, Minisart, 0.2 µm).The filtrate was transferred to an NMR tube with 10% D_2O for NMR experiments. This aqua product was not isolated and used for NMR spectroscopic experiments. ^{1}H NMR (600 MHz): δ (ppm) 8.66 (d, $\underline{H_{2,6}}$, 2H), 8.03 (t, $\underline{H_4}$, 1H), 7.61 (t, $\underline{H_{3,5}}$, 2H), 2.35 (s, C$\underline{H_3}$, ^{3}J (^{195}Pt, ^{1}H) = 13 Hz, 3H). ^{195}Pt NMR (129.4 MHz): $\delta = -1498$ ppm.

Table S1. Fragments observed by MS/MS analysis of parent ion $[Pt(NH_3)(MA)(Py)(5'\text{-}GMP) - H]^+$ ($[\mathbf{1d} - H]^+$) at *m/z* = 684.1228.

Found *m/z*	Assignment	Theoretical *m/z*	Error (ppm)
667.1015	$[\mathbf{1d} - NH_3 - H]^+$	667.0994	3.1
653.0862	$[\mathbf{1d} - MA - H]^+$	653.0838	3.7
636.0607	$[\mathbf{1d} - MA - NH_3 - H]^+$	636.0572	5.5
605.0857	$[\mathbf{1d} - Py - H]^+$	605.0837	3.3
588.0587	$[\mathbf{1d} - Py - NH_3 - H]^+$	588.0572	2.6
574.0427	$[\mathbf{1d} - Py - MA - H]^+$	574.0415	2.1
557.0191	$[\mathbf{1d} - Py - MA - NH_3 - H]^+$ ($[Pt(5'\text{-}GMP) - H]^+$)	557.0150	7.4
522.0272	$[Pt(Py)(G) + H_2PO_4]^+$	522.0255	3.3
460.0108	$[Pt(NH_3)(G) + H_2PO_4]^+$	460.0098	2.2
442.9846	$[Pt(G) + H_2PO_4]^+$	442.9833	2.9
441.0756	$[Pt(NH_3)(Py)(G) - H]^+$	441.0751	1.1
424.0504	$[Pt(Py)(G) - H]^+$	424.0486	4.2
388.0035	$[Pt(NH_3)(Py) + H_2PO_4]^+$	388.0026	2.3
376.0489	$[Pt(MA)(G) - H]^+$	376.0485	1.1
304.0411	$[Pt(MA)(Py) - H]^+$	304.0414	1.0

Table S2. MS/MS analysis for parent ion [Pt(NH$_3$)(MA)(Py)(5'-(8-OH-G)MP) − H]$^+$ ([**1e** − H]$^+$) at *m/z* = 700.1235 and assignment of fragments.

Found *m/z*	Assignment	Theoretical *m/z*	Error (ppm)
682.1123	[**1e** − H$_2$O − H]$^+$	682.1103	2.9
669.0810	[**1e** − MA − H]$^+$	669.0787	3.4
651.0704	[**1e** − MA − H$_2$O − H]$^+$	651.0681	3.5
621.0804	[**1e** − Py − H]$^+$	621.0786	2.9
603.0697	[**1e** − Py − H$_2$O − H]$^+$	603.0681	2.7
586.0447	[**1e** − Py − H$_2$O − NH$_3$ − H]$^+$	586.0415	5.5
572.0278	[**1e** − MA − Py − H$_2$O − H]$^+$	572.0259	3.3
537.0373	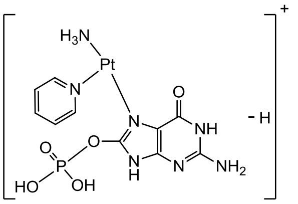	537.0364	1.7
518.0310	Unassigned		
472.0125	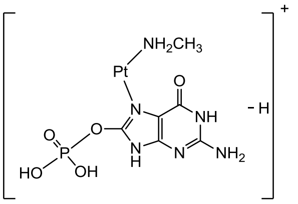	472.0098	5.7
457.0687	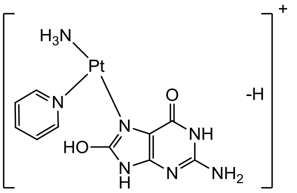	457.0700	2.8
439.0605	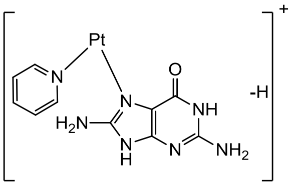	439.0595	2.3
391.0602	Unassigned		
374.0339	Unassigned		
360.3218	Contamination.<br>Also appeared in blank.		
350.5639	[**1e**]$^{2+}$	350.5638	0.3
304.0416	[Pt(MA)(Py) − H]$^+$	304.0408	2.6

Table S3. MS/MS analysis for parent ion [Pt(N_3)(MA)(Py)(5'-GMP)]$^+$ ([**1a**]$^+$) at *m/z* = 710.1205 and assignment of fragments.

Found *m/z*	Assignment	Theoretical *m/z*	Error (ppm)
700.1230	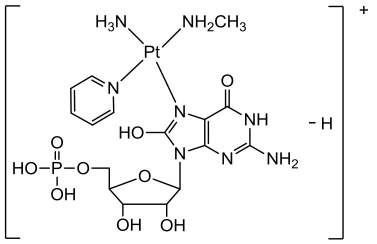	700.1203	3.9
669.0810	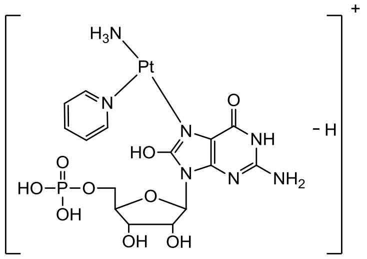	669.0781	4.3
651.0704	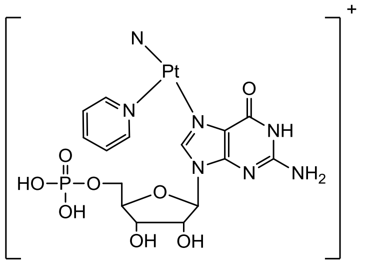 [Pt(N)(Py)(GMP)]$^+$	651.0676	4.3
636.0619	[Pt(Py)(GMP) − H]$^+$	636.0571	7.5
621.0811	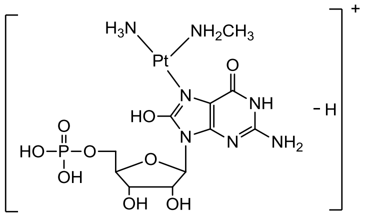	621.0781	4.8
603.0698	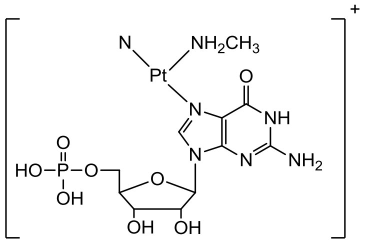 [Pt(N)(MA)(GMP)]$^+$	603.0675	3.8
586.0445	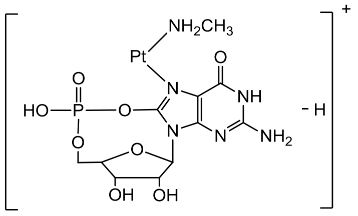	586.0410	6.0

572.0276	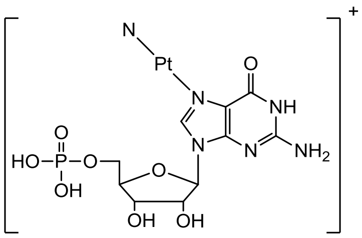 $[Pt(N)(GMP)]^+$	572.0253	4.0
498.1091	$[Pt(N_3)(Py)(MA)(G)]^+$	498.1072	3.8
472.0118	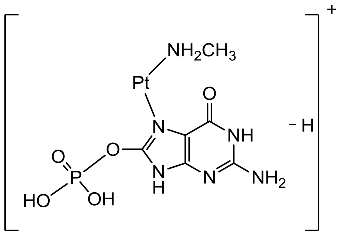	472.0092	5.5
439.0602	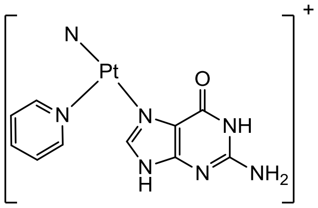 $[Pt(N)(Py)(G)]^+$	439.0589	3.0
391.0596	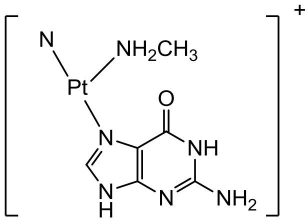 $[Pt(N)(MA)(G)]^+$	391.0589	1.8
374.0336	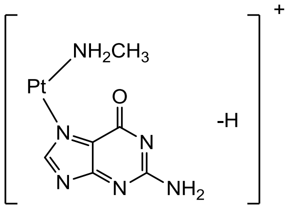	374.0323	3.5
337.9878	$[Pt(N)(MA) + H_3PO_4]^+$	337.9864	4.1
319.0527	$[Pt(N)(MA)(Py)]^+$	319.0517	3.1

Table S4. MS/MS analysis for parent ion [Pt(NH_3)(MA)(Py)(5'-(RedSp)MP) − H]$^+$ ([**1c** − H]$^+$) at *m/z* = 718.1337 and assignment of fragments.

Found *m/z*	Assignment	Theoretical *m/z*	Error (ppm)
700.1227	[**1c** − H_2O − H]$^+$	700.1203	3.4
669.0807	[**1c** − H_2O − MA − H]$^+$	669.0781	3.9
665.0862	[**1c** − 2H_2O − NH_3 − H]$^+$	665.0832	4.5
653.0628	Unspecified		
621.0807	[**1c** − Py − H_2O − H]$^+$	621.0781	4.2
603.0710	[**1c** − Py − 2H_2O − H]$^+$	603.0675	5.8
586.0438	[**1c** − Py − 2H_2O − NH_3 − H]$^+$	586.0410	4.8
472.0117	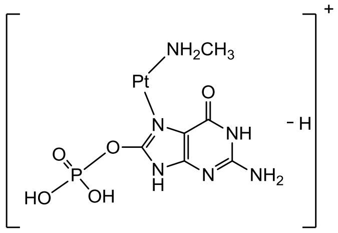	472.0092	5.3
452.0200	Unassigned		
391.0605	Unassigned		
374.0341	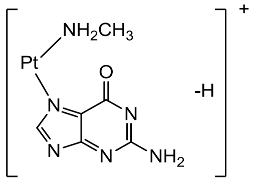	374.0323	4.8

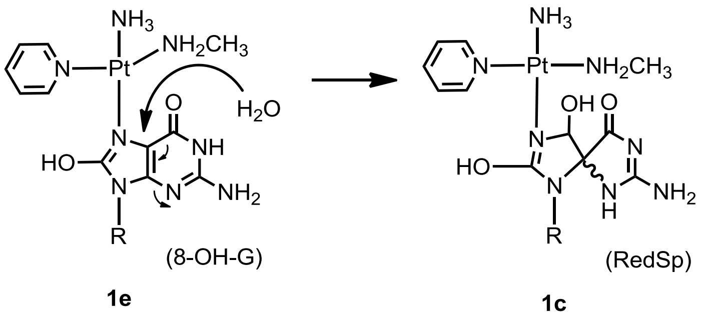

Scheme S1. Pathway for the formation of RedSp (N-formylamidoiminohydantoin).

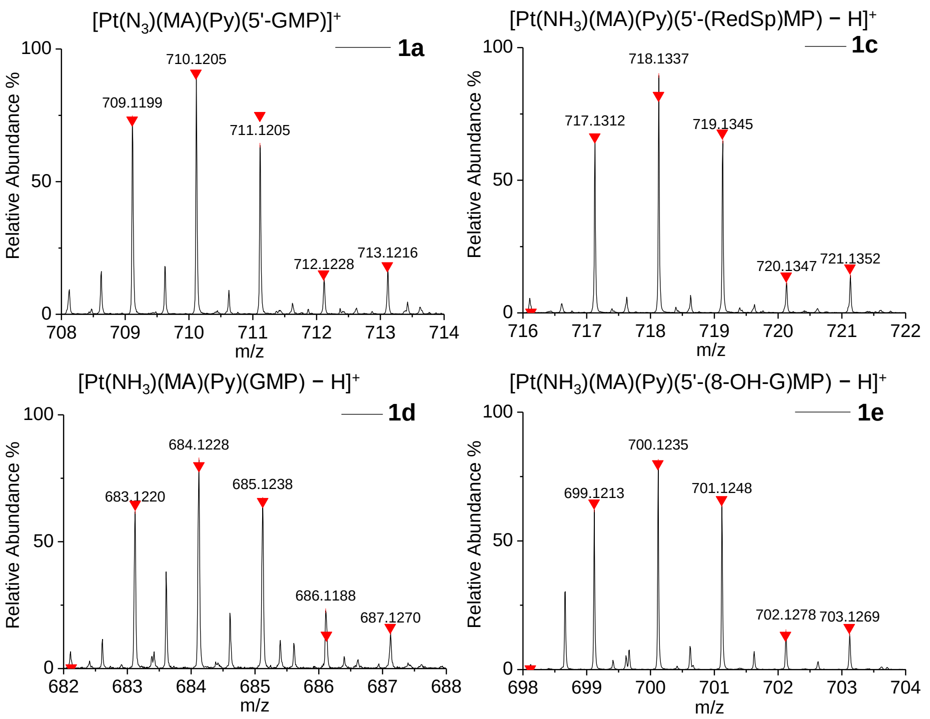

Figure S1. Singly-charged cations found in high resolution mass spectrometry (HR-MS) for species **1a**, **1c**, **1d** and **1e**. The major theoretical isotope distributions of each species (reversed red triangles) are consistent with the peaks found in HR-MS spectra (labelled with numbers). This reaction mixture was analysed by direct infusion ESI-MS.

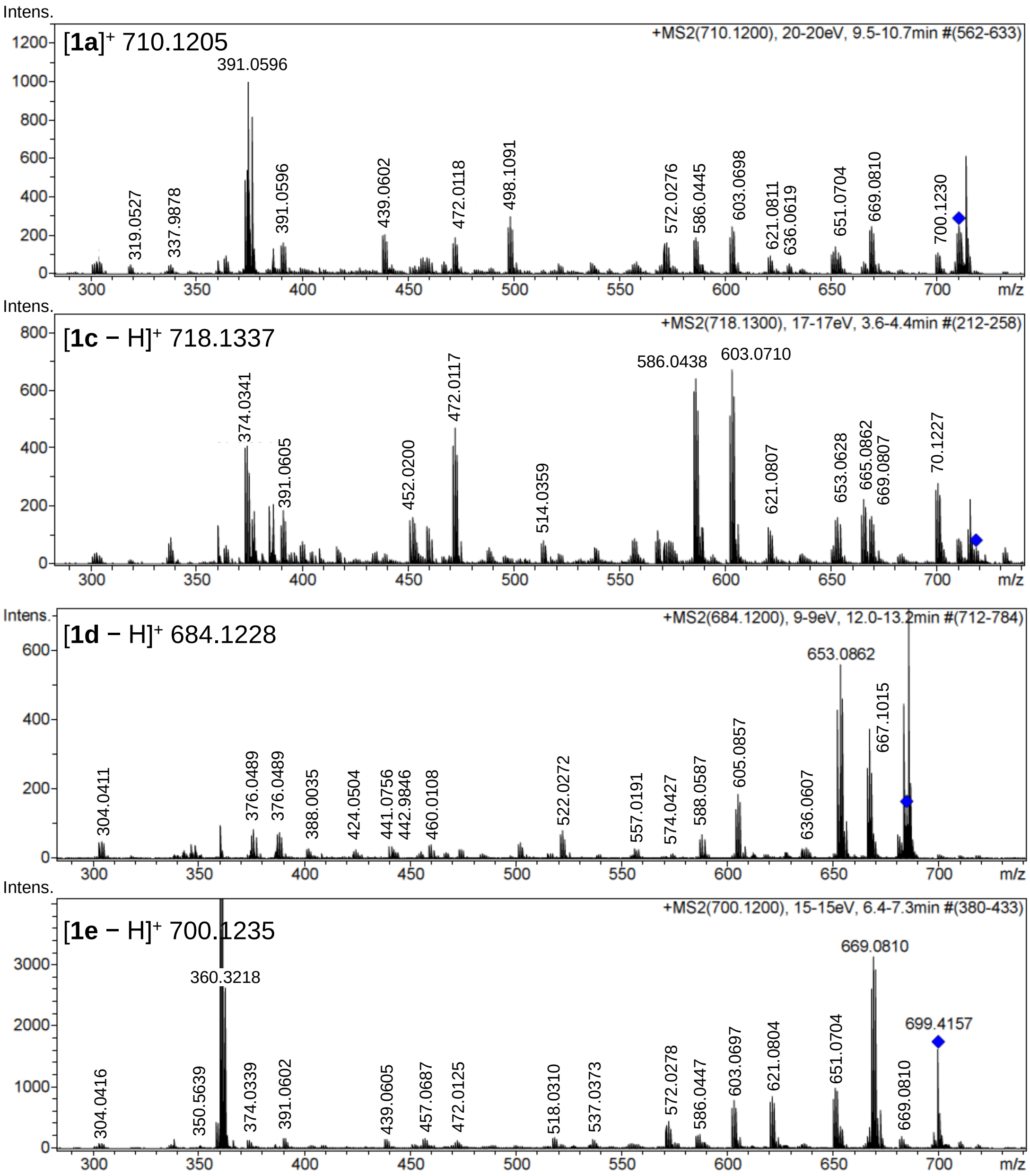

Figure S2. MS/MS spectra (CID) of the parent ions at *m/z* 710.1205 ([**1a**]$^+$), 718.1337 ([**1c** − H]$^+$), 684.1228 ([**1d** − H]$^+$) and 700.1235 ([**1e** − H]$^+$). The assignable peaks and structures are listed in **Table S1** – **S4**.

The cation with *m/z* 684.1228 is assignable to [Pt(NH_3)(MA)(Py)(5'-GMP) − H]$^+$ ([**1d** − H]$^+$) with a mass error of 4.7 ppm between the experimental and theoretical *m/z* (**Table 1**). The MS/MS fragments corresponding to [**1d** − NH_3 − H]$^+$ (667.1015), [**1d** − MA − H]$^+$ (653.0862), [**1d** − Py − H]$^+$ (605.0857) and [Pt(5'-GMP) − H]$^+$ (557.0191) were found (see **Figure S2** and **Table S1**), consistent with assignment as **1d**. The other fragments arising from the MS/MS fragmentation of **1d** are all reasonably assignable to Pt-containing ions with certain combinations of NH_3, MA, Py, GMP, phosphate and sugar moieties.

The cation with *m/z* 700.1235 is assignable to [Pt(NH_3)(MA)(Py)(5'-(8-OH-G)MP) − H] ([**1e** − H]$^+$) with a mass error of 3.7 ppm (**Table 1**). The 5'-(8-OH-G)MP ligand contains an 8-hydroxyguanine (8-OH-G) moiety, which is also known as 8-oxo-guanine (8-oxo-G), one of the most common oxidative DNA lesions.[3] The reason for the oxidation of guanine is elucidated below. The MS/MS analysis of **1e** was consistent with this assignment (**Figure S2** and **Table S2**). Several fragments corresponding to the loss of H_2O were found, e.g., fragment at *m/z* 682.1123, which is assignable to platinum(II) species containing an 8-hydroxyguanosine-5',8-cyclic phosphodiester or 8-hydroxyguanosine-5',3'-cyclic phosphodiester ligand (**Figure S3**).[4] The daughter ions at *m/z* 537.0373 and 472.0125 are assignable to [Pt(NH_3)(Py)(8-OH-G-8-phosphate) − H]$^+$ and [Pt(MA)(8-OH-G-8-phosphate) − H]$^+$, respectively. Other fragments such as [**1e** − MA − H]$^+$ (*m/z* 669.0810), [**1e** − MA − H_2O − H]$^+$ (*m/z* 651.0704), [**1e** − Py − H]$^+$ (*m/z* 621.0804), [**1e** − Py − H_2O − H]$^+$ (*m/z* 603.0697) and [**1e** − Py − H_2O − NH_3 − H]$^+$ (*m/z* 586.0447) further suggested the presence of MA, NH_3 and Py ligands in the molecule.

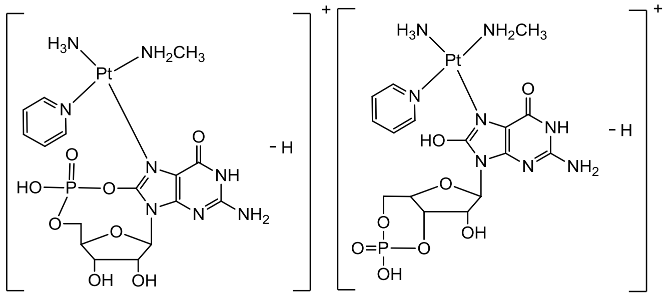

Figure S3. Chemical structures of the possible Pt^{II} fragment at *m/z* = 682.1123 containing a cyclic phosphodiester (8-hydroxyguanosine-5',8-cyclic phosphodiester (left) or 8-hydroxyguanosine-5',3'-cyclic phosphodiester (right)) ligand produced during the MS/MS analysis of **1e**.

The cation with *m/z* 710.1205 is assignable to $[Pt(N_3)(MA)(Py)(5'\text{-}GMP)]^+$ ($[\mathbf{1a}]^+$) with a mass error of 5.8 ppm (**Table 1**). As shown in **Figure S2** and **Table S3**, the MS/MS peaks at *m/z* 651.0704, 572.0276, 439.0602, 391.0596, 337.9878 and 319.0527 correspond to $[Pt(N)(MA)(GMP)]^+$, $[Pt(N)(GMP)]^+$, $[Pt(N)(Py)(G)]^+$, $[Pt(N)(MA)(G)]^+$, $[Pt(N)(MA) + H_3PO_4]^+$ and $[Pt(N)(MA)(Py)]^+$, respectively. These $[Pt(L)N]^+$ species correspond to the loss of N_2 from {Pt-N_3} during MS/MS analysis, as described by **Equation S1**.[5] The oxidation of guanine may also take place during the MS/MS process, forming $[Pt(L - 2H + OH)NH_3]^+$ species, as described in **Equation S2**.[6] The fragments at *m/z* 700.1230, 669.0810 and 621.0811 are assignable to $[Pt(NH_3)(MA)(Py)(5'\text{-}(8\text{-}OH\text{-}G)MP - H)]^+$, $[Pt(NH_3)(Py)(5'\text{-}(8\text{-}OH\text{-}G)MP - H)]^+$ and $[Pt(NH_3)(MA)(5'\text{-}(8\text{-}OH\text{-}G)MP - H)]^+$, respectively.

$$[Pt(L)N_3]^+ \rightarrow [Pt(L)N]^+ + N_2 \qquad \textbf{(S1)}$$

$$[Pt(L)N]^+ + H_2O \rightarrow [Pt(L - 2H + OH)NH_3]^+ \qquad \textbf{(S2)}$$

Other assignable fragments, such as $[Pt(Py)(5'\text{-}GMP) - H]^+$ (636.0619) and $[Pt(N_3)(Py)(MA)(G)]^+$ (498.1091), suggested the presence of MA, 5'-GMP, N_3^- and Py ligands in the molecule. The peaks at *m/z* 586.0445 and 472.0118 were found and assigned as Pt^{II} species containing a cyclic hydroxyguanosine phosphodiester and a 8-OH-G-8-phosphate ligand, respectively (**Table S3**), which also support the formation of 8-hydroxyguanine. Also, the loss of H_2O from the 8-OH-G was found, resulting in a Pt^{II} species with a 2-aminopurin-6-one ligand (dehydrogenated guanine) at *m/z* 374.0336.

The cation at *m/z* 718.1337 is assignable to $[Pt(NH_3)(MA)(Py)(5'\text{-}(RedSp)MP) - H]^+$ ($[\mathbf{1c} - H]^+$) (RedSp = N-formylamidoiminohydantoin) with a mass error of 3.2 ppm (**Table 1**). The product **1c** has a similar structure as **1e** but the 8-OH-G is replaced by RedSp, an hydrolysed 8-OH-G. The hydrolysis of 8-OH-G has been previously reported[3a] and is shown in **Scheme S1**. As **1c** is much more hydrophilic than **1d**, **1e**, and **1a**, the retention time in HPLC for **1c** is much shorter (**Figure 1**). The MS/MS analysis (**Figure S2** and **Table S4**) revealed that **1c** could lose one or two H_2O molecules, which lead to the formation of dehydrated products containing e.g. an 8-cyclic phosphodiester or an 8-OH-G (**Figure S4**). For instance, fragments at *m/z* 700.1227, 669.0807, 665.0862, 621.0807, 603.0710 and 586.0438 are assignable to $[\mathbf{1c} - H_2O - H]^+$, $[\mathbf{1c} - H_2O - MA - H]^+$, $[\mathbf{1c} - 2H_2O - NH_3 - H]^+$, $[\mathbf{1c} - Py - H_2O -$

H]$^+$, [**1c** − Py − $2H_2O$ − H]$^+$ and [**1c** − Py − $2H_2O$ − NH_3 − H]$^+$, respectively. These fragments also suggest the presence of MA, MH_3 and Py groups in the molecule. The loss of H_2O from 8-OH-G (*m/z* 374.0341) was again found here.

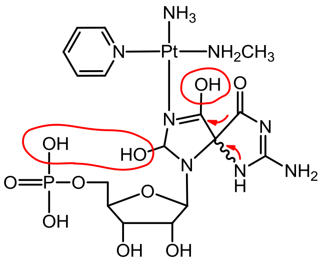

Figure S4. Possible dehydration positions on **1c** in MS/MS, indicated with circles and arrows.

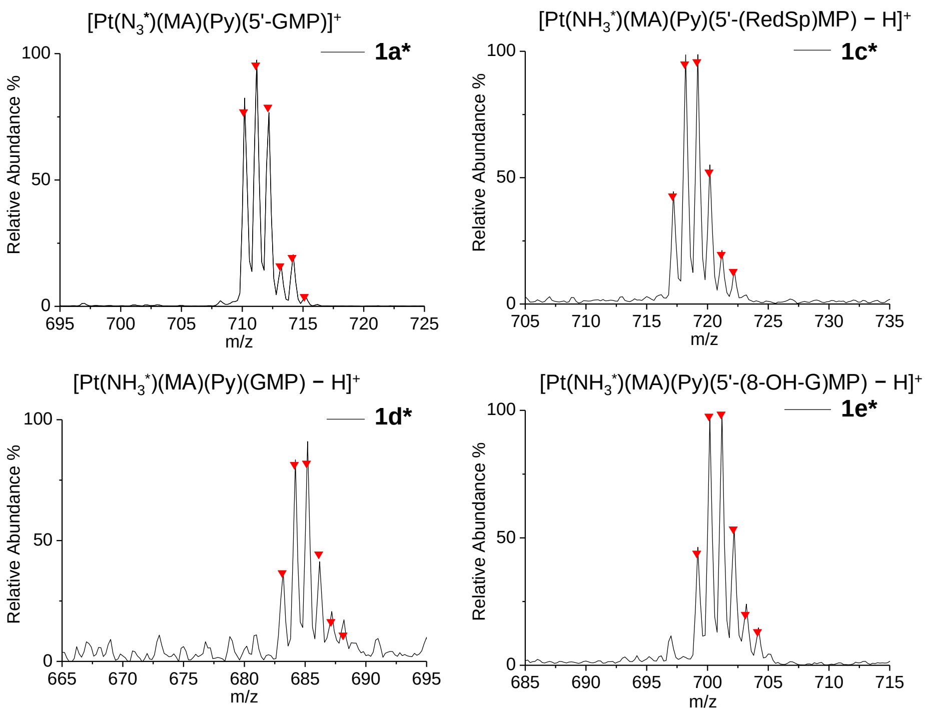

Figure S5. Mass spectra for singly-charged cations of **1a***, **1c***, **1d*** and **1e*** obtained by LC-MS (HCT-Ultra). The major calculated isotope distributions for each ion are labelled with red triangles.

The ESI-MS spectra for **1a***, **1c*** **1d*** and **1e*** are shown in **Figure S5**. If the molecule contains the intact end-labelled N_3^{*-} ligand, the *m/z* of each peak in the isotopic distribution should be 1 Da larger than that of the unlabelled molecule. If the molecule contains an NH_3 group converted from the end-labelled N_3^{*-} ligand, the N atom should be a 50%/50% mixture of ^{15}N and ^{14}N. Therefore, the resultant isotope distributions should be a 50%/50% mixture of M and M + 1. The cations in **Figure S5** are assigned as $[Pt(N_3^*)(MA)(Py)(5'\text{-GMP})]^+$ ([**1a***]$^+$), $[Pt(NH_3^*)(MA)(Py)(5'\text{-(RedSp)MP}) - H]^+$ ([**1c*** − H]$^+$]), $[Pt(NH_3^*)(MA)(Py)(GMP) - H]^+$ ([**1d*** − H]$^+$) and $[Pt(NH_3^*)(MA)(Py)(5'\text{-(8-OH-G)MP}) - H]^+$ ([**1e*** − H]$^+$).

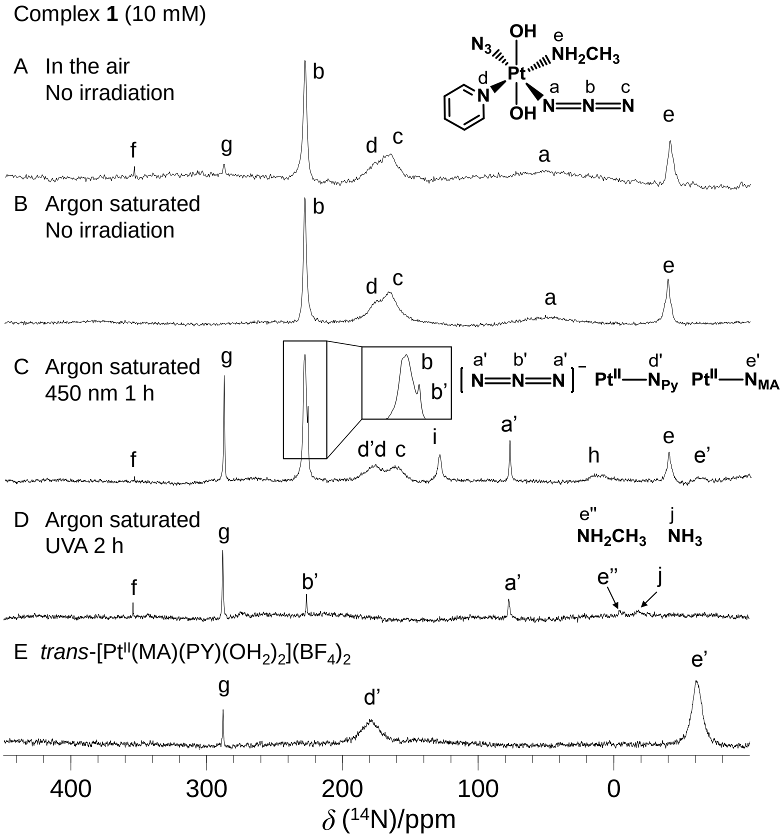

Peak	δ/ppm	Assignment
**a**	70 – 80	Pt^{IV}–*N*NN
**b**	228	Pt^{IV}–N*N*N
**c**	165	Pt^{IV}–NN*N*
**a'**	78	*N*N*N*$^{-}$
**b'**	226	N*N*N^{-}
**d**	174	Pt^{IV}–N_{Py}
**d'**	179	Pt^{II}–N_{Py}
**e**	−39	Pt^{IV}–N_{MA}
**e'**	60	Pt^{II}–N_{MA}
**e"**	−3	NH_2CH_3
**f**	354	NO_3^{-}
**g**	288	N_2 gas
**h**	~15	n.a.
**i**	130	n.a.
**j**	−18	NH_3

n.a. = not assigned.

Figure S6. ^{14}N NMR (43.4 MHz) spectrum of **1** (10 mM in 90% H_2O:10% D_2O, pH adjusted to 7.4 ± 0.5) with the assignments of signals listed. (**A**) Sample prepared in air and kept in the dark; (**B**) sample **A** saturated with argon, (**C**) sample **B** irradiated at 450 nm for 60 min; (**D**) sample **B** irradiated with UVA for 2 h; (**E**) *trans*-[Pt(MA)(Py)$(OH_2)_2$]$(BF_4)_2$ (**1g**) (10 mM in 90% H_2O:10% D_2O).

^{14}N NMR spectroscopy was used to probe the change in the N_3^{-} ligands and the other N atoms during the photolysis of **1** (**Figure S6**). The assignments of the signals were made according to the ^{14}N NMR results of the authentic samples or published data for related complexes.[7] First, the 1D ^{14}N NMR spectrum of **1** in 90% H_2O and 10% D_2O was recorded (**Figure S6A**). The signal at 288 ppm (peak **g**) was assignable to dissolved nitrogen gas, which disappeared after saturating the solution with argon (**Figure S6B**). Peaks **b** (228 ppm), **c** (165 ppm) and the broad signal **a** (~ 80 ppm) correspond to the central, unbound terminal and Pt-bound terminal N atoms, respectively, of coordinated N_3^{-}. The resonance for Pt^{IV}-bound-N (**a**) in the azido ligand is exceptionally broad due to an highly asymmetric electric field gradient at the ^{14}N nucleus.[7c] Peaks **d** (174 ppm) and **e** (−39 ppm) correspond to the N atoms of the Py and MA, respectively.

After irradiation at 450 nm (50 mW/cm^2) at 298 K for 1 hour, new ^{14}N NMR spectroscopic signals appeared (**Figure S6C**). Peaks **b'** (226 ppm) and **a'** (78 ppm) are assignable to the terminal and central nitrogen atoms of free azide (N_3^-). This assignment was consistent with the ^{14}N NMR spectrum of NaN_3 in D_2O and other related compounds.[7a,8] Other new peaks **d'** (δ = −179 ppm) and **e'** (δ = −60 ppm) are assigned as the Pt^{II}-coordinated Py and MA ligands, respectively. These assignments were consistent with the ^{14}N NMR spectrum of the di-aqua species of *trans*-[Pt(MA)(Py)(OH_2)$_2$](BF_4)$_2$ (**1g**) which was synthesized independently (**Figure S6E**). Peaks **b** (228 ppm) and **c** (165 ppm) for the coordinated N_3^- ligand of complex **1** were still present, but with reduced intensities. The signals **d** and **d'** were both very broad due to the effect of quadrupolar ^{14}N, but they could still be distinguished in **Figure S6C**. Remarkably, the sharp peak **g** corresponding to nitrogen gas (N_2) was also observed. The release of N_2 gas is consistent with the GC-MS results (**Figure S9**). The N_2 gas may be released directly from {Pt-N_3} and may also be formed from the combination of the N_3• radicals generated in this photoreaction.[9] The release of N_3• radicals was supported by EPR analysis (**Figure S7**). Peak **f** is assigned as free nitrate NO_3^- in solution, arising as a side-product of the photodecomposition of the azido ligand.[7a,7b] The other new peaks **h** and **i** were not identified.

After irradiation of an argon-saturated sample of **1** with UVA (3.46 mW/cm^2) for 2 hours, gas bubbles and significant amounts of precipitate were observed. Almost no peaks assignable to coordinated azide were detected, and intriguingly, the signals for coordinated Py and MA were not observed either (**Figure S6D**). It is likely that most of the Py and MA ligands were present in the precipitate. Three new signals **b'** (226 ppm), **c'** (78 ppm) and **g** (δ = 288 ppm) were again assignable as the terminal and central nitrogen atoms of free azide (N_3^-) and nitrogen gas, respectively. A very small amount of free MA ($\underline{N}H_2CH_3$) **e"** (δ = −3 ppm) and free ammonia $\underline{N}H_3$ **j** (δ = −18 ppm) was detected. These signals were confirmed by ^{14}N NMR experiments for authentic samples and also by data in the literature.[7b] Free MA is from the photo-disassociation of Pt-N(MA) bond. It was surprising to find the signal of free NH_3. Previous reports suggest that upon irradiation with light, N_2 gas may be released directly from the {Pt-N_3}, forming a nitrene intermediate {Pt-N}.[10] This intermediate can convert to {Pt-NH_3} (**Figure S5**), followed by photo-dissociation to give free NH_3.[6-7,11] Free N_3^- anions can also undergo this series of photoreactions and give rise to free NH_3.[12]

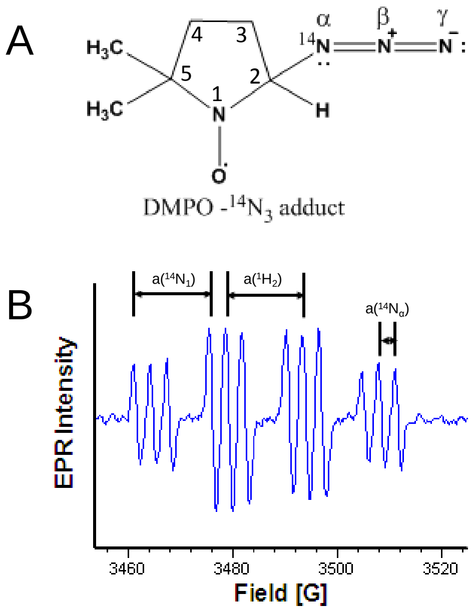

Figure S7. (**A**) Structure of spin adduct DMPO–$^{14}N_3$; (**B**) EPR spectrum of irradiated (450 nm, 10 mW/cm^2, 5 min) sample of complex **1** (5 mM) with DMPO (10 mM) in deionised water (293 K).

An unambiguous way to identify the N_3• radicals is electron paramagnetic resonance (EPR) using 5,5-dimethyl-pyrroline-N-Oxide (DMPO) as the spin-trap. The hyperfine splitting patterns of the EPR signal and the g-factor are the “finger print” of trapped radicals. Complex **1** (5 mM) and 2 mol equiv of DMPO in aqueous solution was irradiated at 450 nm (50 mW/cm^2) at 293 K. The resulting EPR spectrum which is compatible with the adduct DMPO–$^{14}N_3$ is shown in **Figure S7A**. The simulation of hyperfine splittings consists of a quartet of triplets (1:1:1:2:2:2:2:2:2:1:1:1). This results from overlap of the hyperfine splitting constants (HFSC) of the proton ($^1H_2$) and the nitroxyl nitrogen ($^{14}N_1$), with further splitting by the azide nitrogen ($^{14}N_\alpha$). The EPR signal was observed in the first spectrum recorded within 5 min (**Figure S7B**), and is in good agreement with the simulation and previously reported data for trapped N_3•.[9b,13] The hyperfine splitting constants (HFSC) were: a($^{14}N_1$) = 14.4 G, a($^1H_2$) = 14.6 G, a($^{14}N_\alpha$) = 3.2 G and the g-factor = 2.011. The decay half-time of DMPO–$^{14}N_3$ spin adduct was *ca*. 25 min.

The hydroxyl radical OH• can also be captured by DMPO, and the EPR signal of DMPO-OH should have the hyperfine splitting pattern of 1:2:2:1.[13a,14] However, in

this work, no signal corresponding to DMPO-OH was found. Therefore, OH• radicals may not be generated in the photolysis of complex **1** under the conditions used, or their lifetime may be very short. An additional experiment was conducted on complex **1** and DMPO in the presence of 2 mol equiv of 5'-GMP with other conditions being identical to these described above. The EPR spectrum was identical to that shown in **Figure S7B**, indicating that excess 5'-GMP did not affect the generation or trapping of N_3• radicals, or that the spin-trap DMPO reacts with the N_3• radical faster than 5'-GMP.

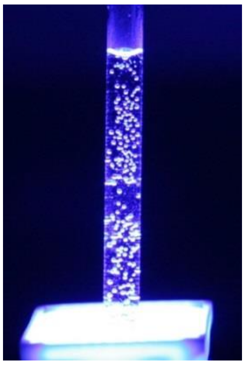

Figure S8. Gas bubbles observed in an NMR tube during irradiation of a D_2O solution of **1** (3.9 mM)/5'-GMP (7.8 mM) with a 450 nm LED over 30 min (298 K).

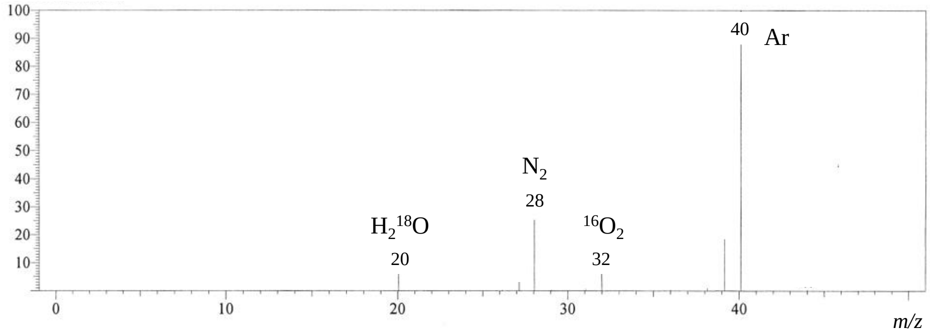

Figure S9. GC-MS spectrum for the gas phase from the photolysis of **1** in ^{18}O-water upon irradiation with UVA (λ_{max} = 365 nm, 4 mW/cm^2). Peaks for $^{16}O_2$ (*m/z* = 32), N_2 (*m/z* = 28), $H_2{}^{18}O$ (*m/z* = 20) and argon (*m/z* = 40) were observed.

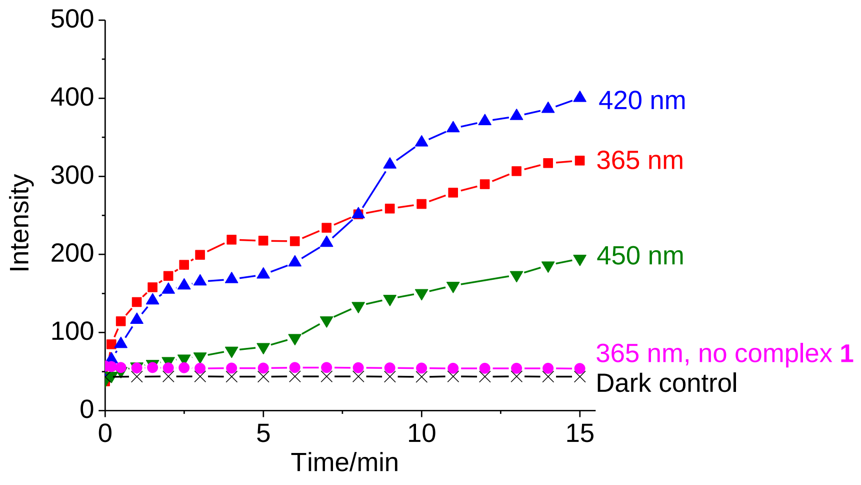

Figure S10. The time dependent intensity of the fluorescence (λ_{ex} = 504 nm, λ_{em} = 525 nm) of **1** (50 µM) and SOSG (1 µM) in H_2O (3% MeOH) upon irradiation at various wavelengths. T = 290 – 295 K. ■, λ_{irr} = 365 nm, 21 µW/cm^2; ▲, λ_{irr} = 420 nm, 850 µW/cm^2; ▼, λ_{irr} = 450 nm, 800 µW/cm^2; ×, dark control; ●, no complex **1** added, λ_{irr} = 365 nm. All the data points were the average of 2 – 4 independent experiments.

The efficiency of $^1O_2$ generation by different irradiation wavelengths was compared. As depicted in **Figure S10**, fluorescence was also observed upon irradiation at 420 or 450 nm. It is notable that the power densities of 420 and 450 nm light are higher than that of 365 nm, so the dose-dependent efficiency of generating $^1O_2$ upon irradiation with shorter wavelengths was still higher than that with the longer wavelengths. The pH of reaction mixture decreased from *ca.* 7 to 6, as measured by a pH paper, which excludes the activation of SOSG at alkaline pH.[15] The dark control experiment for the solution of complex **1**/SOSG (50 µM/1 µM) showed very low intensity of fluorescence. Low fluorescence was also observed in the absence of complex **1**.

References

[1] R. K. Harris, E. D. Becker, S. M. C. de Menezes, R. Goodfellow, P. Granger, *Pure Appl. Chem.* **2001**, *73*, 1795-1818.

[2] Y. Zhao, J. A. Woods, N. J. Farrer, K. S. Robinson, J. Pracharova, J. Kasparkova, O. Novakova, H. Li, L. Salassa, A. M. Pizarro, G. J. Clarkson, L. Song, V. Brabec, P. J. Sadler, *Chem. Eur. J.* **2013**, *19*, 9578-9591.

[3] a) G. Pratviel, B. Meunier, *Chem. Eur. J.* **2006**, *12*, 6018-6030; b) S. Kanvah, J. Joseph, G. B. Schuster, R. N. Barnett, C. L. Cleveland, U. Landman, *Acc. Chem. Res.* **2010**, *43*, 280-287.

[4] S. Choi, R. B. Cooley, A. S. Hakemian, Y. C. Larrabee, R. C. Bunt, S. D. Maupas, J. G. Muller, C. J. Burrows, *J. Am. Chem. Soc.* **2004**, *126*, 591-598.

[5] R. A. J. O'Hair, in *Reactive Intermediates - MS Investigations in Solution* (Ed.: L. S. Santos), Wiley-VCH Verlag GmbH & Co. KGaA, Weinheim, **2010**, pp. 199-227.

[6] S. Wee, J. M. White, W. D. McFadyen, R. A. J. Hair, *Aust. J. Chem.* **2003**, *56*, 1201-1207.

[7] a) H. I. A. Phillips, L. Ronconi, P. J. Sadler, *Chem. Eur. J.* **2009**, *15*, 1588-1596; b) L. Ronconi, P. J. Sadler, *Dalton Trans.* **2011**, *40*, 262-268; c) N. J. Farrer, P. Gierth, P. J. Sadler, *Chem. Eur. J.* **2011**, *17*, 12059-12066.

[8] J. Kent, *J. Chem. Phys.* **1966**, *44*, 3530.

[9] a) S. J. David, R. D. Coombe, *J. Phys. Chem.* **1986**, *90*, 3260-3263; b) J. S. Butler, J. A. Woods, N. J. Farrer, M. E. Newton, P. J. Sadler, *J. Am. Chem. Soc.* **2012**, *134*, 16508-16511.

[10] L. Ronconi, P. J. Sadler, *Chem. Commun.* **2008**, 235-237.

[11] J. Šima, *Coord. Chem. Rev.* **2006**, *250*, 2325-2334.

[12] V. R. P. Verneker, M. Blais, *J. Phys. Chem.* **1968**, *72*, 774-778.

[13] a) W. Kremers, A. Singh, *Can. J. Chem.* **1980**, *58*, 1592-1595; b) B. Kalyanaraman, E. G. Janzen, R. P. Mason, *J. Biol. Chem.* **1985**, *260*, 4003-4006.

[14] S. Luanpitpong, U. Nimmannit, P. Chanvorachote, S. Leonard, V. Pongrakhananon, L. Wang, Y. Rojanasakul, *Apoptosis* **2011**, *16*, 769-782.

[15] Molecular Probes, Product information for SOSG, http://probes.invitrogen.com/media/pis/mp36002.pdf?id=mp36002, accessed on 20 Aug, 2013.
